# Voluntary postural sway control and mobility in adults with low back pain

**DOI:** 10.3389/fnins.2023.1285747

**Published:** 2024-01-03

**Authors:** Zhengquan Chen, Oren Tirosh, Jia Han, Roger David Adams, Doa El-Ansary, Adrian Pranata

**Affiliations:** ^1^Shanghai Yangpu District Mental Health Center, Shanghai University of Medicine & Health Sciences, Shanghai, China; ^2^Department of Nursing and Allied Health, School of Health Sciences, Swinburne University of Technology, Hawthorn, VIC, Australia; ^3^School of Health and Biomedical Sciences, RMIT University, Melbourne, VIC, Australia; ^4^College of Rehabilitation Sciences, Shanghai University of Medicine & Health Sciences, Shanghai, China; ^5^Research Institute for Sport and Exercise, University of Canberra, Canberra, ACT, Australia; ^6^Department of Surgery, Melbourne Medical School, Melbourne, VIC, Australia

**Keywords:** proprioception, low back pain, postural control, mobility, somatosensory perception

## Abstract

**Introduction:**

Low back pain (LBP) is associated with altered somatosensory perception, which is involved in both involuntary and voluntary control of posture. Currently, there is a lack of methods and tools for assessing somatosensory acuity in patients with LBP. The purpose of this study was (1) to assess the reliability of the sway discrimination apparatus (SwayDA) (2) to evaluate the differences in somatosensory acuity between patients with LBP and pain-free individuals, and (3) to examine relationships between somatosensory acuity, severity of LBP, and mobility in patients with LBP.

**Methods:**

Twenty participants (10 patients with LBP and 10 matched asymptomatic controls) were recruited in a test–retest reliability test. Another 56 participants were recruited for this study with 28 individuals presenting with LBP and a further twenty-eight being asymptomatic. The SwayDA was custom-built to measure somatosensory perception during voluntary anterior–posterior (SwayDA-AP), medial-lateral to the dominant side (SwayDA-ML-D), and non-dominant side (SwayDA-ML-ND) postural sway control. Participants also completed mobility tests, including 10 times and 1-min sit-to-stand tests (10-STS, 1 m-STS). The area under the receiver operating characteristic curve (AUC) was calculated to quantify somatosensory acuity in discriminating different voluntary postural sway extents.

**Results:**

The ICC (2.1) for the SwayDA-AP, SwayDA-ML-D, and SwayDA-ML-ND were 0.741, 0.717, and 0.805 with MDC_95_ 0.071, 0.043, and 0.050. Patients with LBP demonstrated significantly lower SwayDA scores (t_SwayDA-AP_ = −2.142, *p* = 0.037; t_SwayDA-ML-D_ = -2.266, *p* = 0.027) than asymptomatic controls. The AUC values of the SwayDA-AP test were significantly correlated with ODI (r_SwayDA-AP-ODI_ = −0.391, *p* = 0.039). Performances on the 1 m-STS and the 10-STS were significantly correlated with the AUC scores from all the SwayDA tests (−0.513 ≤ r ≤ 0.441, all *p* < 0.05).

**Discussion:**

The SwayDA tests evaluated showed acceptable reliability in assessing somatosensory acuity during voluntary postural sway. Somatosensory acuity was diminished in patients with LBP compared to asymptomatic controls. In patients with LBP, lower somatosensory acuity was associated with increased LBP-related disability. Future research could focus on investigating the factors contributing to the decreased somatosensory perception and mobility in individuals with LBP.

## 1 Introduction

Low back pain (LBP) is a common musculoskeletal condition that is often accompanied by decreased sensory, cognitive, and mobility function, presenting an economic health burden worldwide (Chiarotto and Koes, 2022). The direct and indirect economic burden for LBP ranged from US$19.6 to $118.8 billion in the USA in 2008 (Dagenais et al., 2008), and the number was approximately US$ 4.7 billion (AU$ 9.17 billion) in Australia (Walker et al., 2003). It is estimated that the burden from LBP-related costs and disability will expand further in the future (GBD, 2021). The point prevalence standardized by age was estimated at 7.0 to 18.3% [5; 6]. Although the prevalence of LBP among young adults aged 20–29 years old was the lowest, the prevalence in this age group was still estimated to be as high as 10–15% (Hoy et al., 2012; Chen et al., 2022). LBP inevitably affects the social participation resulting in absences from education and training, work, and participation in sport (Hartvigsen et al., 2018).

Although LBP is a condition that presents with primary symptoms affecting the lower back and/or legs, it may also affect the control of involuntary postural sway in static stance impacting the body globally (Berenshteyn et al., 2019). From the perspective of the inverted pendulum model of the human body (Reeves et al., 2011), the control parameters of involuntary postural sway in the sagittal plane and coronal plane reflect the postural control ability of the individual in involuntary activities, where a smaller amplitude of postural sway and a slower speed represent a higher level of postural sway control ability (King et al., 2019). Mohammadi et al. (2021) reported that the sagittal plane velocity was a sensitive parameter related to decreased somatosensory perception and postural control ability in patients with LBP (Mohammadi et al., 2021). Further, postural sway control in the coronal plane has been demonstrated to be more challenging and energy-consuming than sway in the sagittal plane (Slobounov et al., 2008). Accordingly, patients with LBP may also have decreased postural control ability for mediolateral sway, likely due to pain. Evidence from a systematic review demonstrated that the amplitude of mediolateral sway was increased in patients with LBP in most of the included studies that involved mediolateral postural sway control (Ruhe et al., 2011). Further evidence has highlighted the sensitivity and vulnerability of mediolateral postural sway control ability when somatosensory perception was disturbed (Berenshteyn et al., 2019).

Notably, there is currently no consensus on the evaluation of voluntary postural sway control ability. However, some studies attempted to describe the changes in voluntary postural control from the perspective of kinematics and kinetics, and the results have demonstrated that patients with LBP had an overall decline in both sagittal and coronal planes during voluntary postural control tasks (Cullen, 2012; Ayhan et al., 2016; Mokhtarinia et al., 2016). Ayhan et al. (2016) observed the performance of patients with LBP in voluntary postural control tasks using a commercialized device measuring postography, including limit of stability test, rhythmic weight shift test, and toes-up and toes-down tests (Ayhan et al., 2016). The results of this study showed that patients with LBP had poor orientation and delayed movement initiation, which weakened their performance in voluntary postural control (Ayhan et al., 2016). The motion capture system has also been applied to quantitatively evaluate the performance of different body segments during voluntary postural control. In the sagittal plane, patients with LBP showed worse coordination between kinematic chains (lumbar spine-pelvis and thigh-pelvis) than asymptomatic controls, especially in asymmetric movements during repeated trunk flexion-extension tasks (Mokhtarinia et al., 2016). A systematic review of gait changes showed that patients with LBP had significantly slower walking speeds and shorter stride lengths, which were related to poor control of weight shifting in the coronal plane (Smith et al., 2022). However, studies on kinematics and kinetics rely on laboratory-based equipment with demanding requirements for the environment and technicians.

Voluntary postural control requires a complex interplay of somatosensory information and motor responses. The ability to perceive the amplitude of weight shifting is integral to maintaining stability and adapting to voluntary postural control tasks and environmental demands (Sibley et al., 2015; Brumagne et al., 2019). It is conceivable that alteration of voluntary postural control is associated with decreased somatosensory perception of voluntary postural sway extents in patients with LBP. For patients with LBP, however, to the authors’ knowledge, there is no identified method for quantifying somatosensory acuity during voluntary postural sway.

Han et al. (2016) proposed a psychophysical method of assessing somatosensory acuity through analysis of absolute judgments of different voluntary movement extents (Han et al., 2016). Some clinical assessments using psychophysical theory, such as the Ankle Inversion Discrimination Apparatus-Walking and Landing tests (Yu et al., 2021; Shao et al., 2023), have been widely used in sports injury prevention and performance enhancement. Chen and colleagues also designed a voluntary postural sway discrimination apparatus (SwayDA) to quantify somatosensory acuity during voluntary postural sway in the coronal plane and tested it with older people (Chen et al., 2019).

In this study, the SwayDA has been adapted to assess somatosensory acuity during voluntary anteroposterior and mediolateral sway. This modification was motivated by the overall decrease in voluntary postural control in both sagittal and coronal planes among patients with LBP. Therefore, the purpose of this study is threefold: firstly, to assess the test–retest reliability of the modified SwayDA; secondly, to evaluate impairment of somatosensory acuity in patients with LBP compared to asymptomatic individuals; and thirdly, to explore the relationship between somatosensory acuity and performance in mobility tasks. It is hypothesized that the SwayDA tests in the coronal and sagittal planes will have good test–retest reliability; that patients with LBP will show decreased somatosensory acuity, and that there will be a positive association between the changes in somatosensory acuity and mobility.

## 2 Materials and methods

### 2.1 Participants

#### 2.1.1 Part 1: Reliability

Twenty participants, including those with LBP (*n* = 10) and asymptomatic individuals (*n* = 10), were recruited for the test–retest reliability assessment of a purpose-built device, SwayDA.

#### 2.1.2 Part 2: Validity

With respect to known-groups validity and convergent validity of the SwayDA tests a further 56 participants were invited to the study.

All participants involved in this study were adults aged over 18 years (mean age ± SD, 19.9 ± 2.5). In the control group participants were required to be asymptomatic, pain-free during the time of testing, with no history of LBP. The inclusion criteria for LBP participants were self-reported non-specific pain between T12-S1, with or without associated leg symptoms (Knezevic et al., 2021) after the acute phase (8 weeks).

Participants were excluded if they: (1) reported a history of major psychiatric and psychological illness (e.g., severe depression); (2) were diagnosed with visual disorders, vestibular disorders, or neurological disorders that would affect balance (e.g., multiple sclerosis); (3) had spine or lower limb injuries or surgery in the past 6 months; (e.g., ankle sprain, fracture); (4) were currently pregnant or < 3 months post-partum; (5) took medications that impact balance; (6) received physiotherapy for LBP in the past 6 months. This study has been approved by Swinburne University of Technology Human Research Ethics Committee (Ethics ID: 20225788–11,032). Signed informed consent was obtained from participants before they participated in the study.

### 2.2 Design of a novel apparatus

The design of the SwayDA was based on the concept of a testing posture designed to maximize ecological validity, and a psychophysical approach to data analysis (Han et al., 2016). Anterior–posterior (AP) and medial-lateral (ML) sways were the two main components of postural sway control examined (Ruhe et al., 2011; Knox et al., 2018; Berenshteyn et al., 2019).

**Figure 1:**
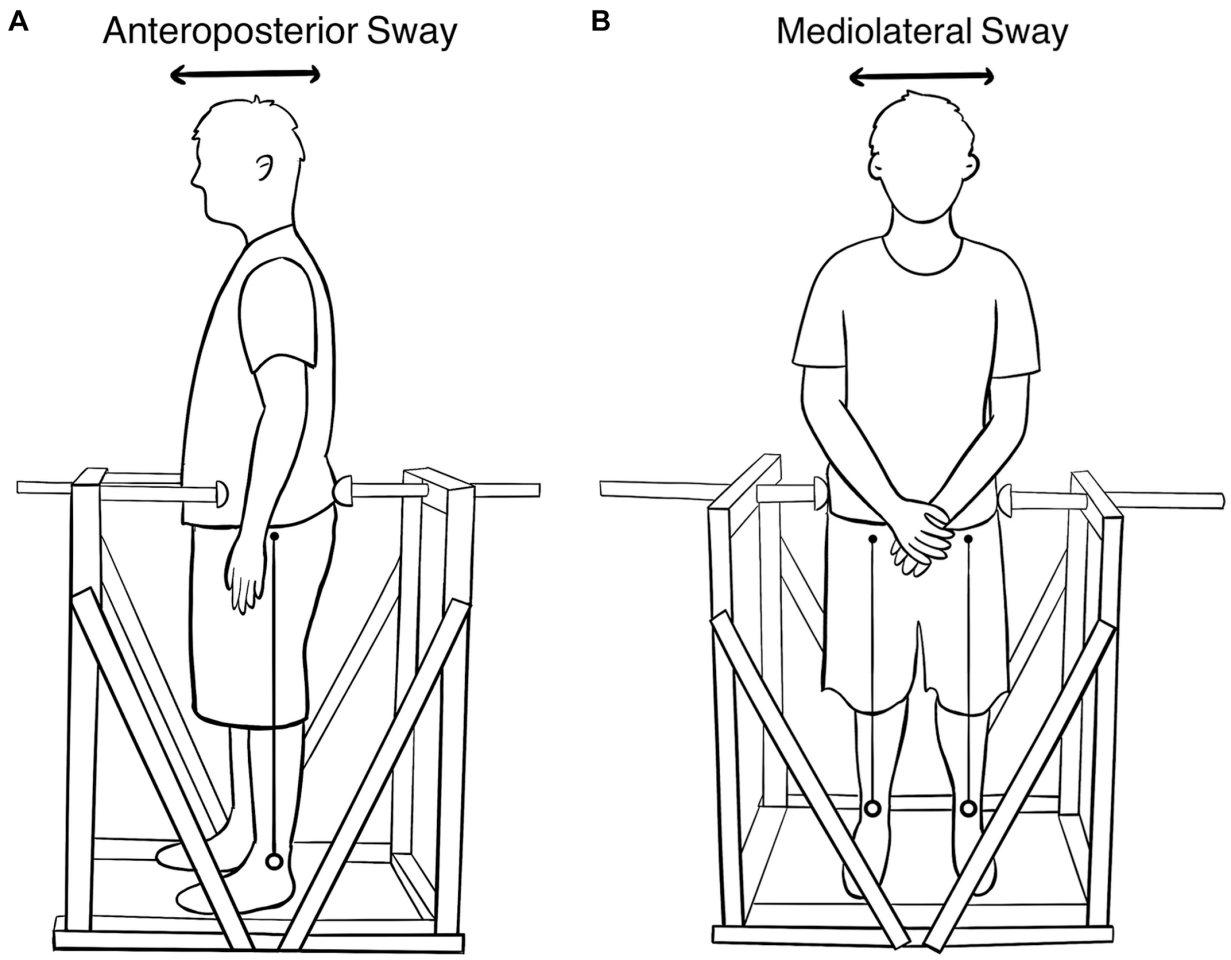
**(A)** The SwayDA-AP test. The stop on the left is the contact point of the anterior superior iliac spine, and the rear stop is adjustable. **(B)** The SwayDA-ML test. The stop on the left is the contact point of the greater trochanter of the femur. The right-side stop is adjustable.

The SwayDA (Figure 1) is 100 cm × 60 cm × 120 cm (length, width, height). It comprises a standing platform with two adjustable hemispherical wooden stops attached to fixed vertical wooden frames, each designed for a specific AP and ML voluntary sway directions. To prevent excessive movements to both sides, the frames and platform were reinforced with wooden boards. Wooden stops were provided, offering a set of four predetermined AP or ML sway extents. Additionally, a movable wooden stop on the other side was used to indicate a neutral standing position, which could be adjusted according to the individual’s body size.

### 2.3 Testing procedure

#### 2.3.1 The SwayDA tests

The SwayDA tests utilized the psychophysical method of absolute judgment and non-parametric signal detection theory-based analysis (Hautus et al., 2022) to measure the participant’s accuracy in discriminating a set of sway extents in the sagittal and coronal planes. Participants were required to wear sports clothes, stand on the platform without shoes, and position their heels in line with the sciatic tubercles. In the SwayDA-AP test (Figure 1A), participants were instructed to face the movable stop on one side and look straight ahead without a fixed target (Moraes et al., 2016). Four sway amplitudes were pre-set on the movable stop: Position 1 was set 2 cm in front of the neutral standing position, and Positions 2, 3, and 4 represented sway distances of 2.5 cm, 3 cm, and 3.5 cm, respectively, in the anteroposterior direction.

Before data collection, a familiarization session was conducted, consisting of 3 rounds of practice from Position 1 to Position 4 sequentially (a total of 12 practice trials). The anteroposterior sway pattern resembles the movement of an inverted pendulum, where participants initiated sway from their ankles while keeping their hips and knees fixed. Upon their anterior superior iliac spine contacting the wooden stop, participants were required to sway back to the neutral starting position, and then respond with a position number. During the testing session, the 4 sway amplitudes were presented 10 times in a random order, resulting in a total of 40 trials. In each trial, participants were asked to make a numeric judgment (Position 1, 2, 3, or 4) regarding the extent of their sway, as soon as they returned to the starting position.

The procedure of the SwayDA-ML test was similar to that of the SwayDA-AP test. Specifically, participants were instructed to voluntarily sway their body side-to-side (not hip tilting) from the neutral stand position toward the hemispherical stop in the SwayDA-ML test (Figure 1B). There were two sessions in the SwayDA-ML test, one involving lateral sway to the dominant side (the SwayDA-ML-D test) and the other to the non-dominant side (the SwayDA-ML-ND test).

Each trial in both the SwayDA-AP and SwayDA-ML tests took approximately 3 s to operate the movable stop to a specific sway extent and record the participant’s response. The estimated duration for each test session was approximately 5 min. The testing sequence for the SwayDA-AP and SwayDA-ML tests was randomized. Furthermore, participants were provided with a one-minute break before the start of each test session to prevent fatigue. Detailed testing procedures of the SwayDA tests were provided in Supplementary material 1.

#### 2.3.2 Reliability

To assess test–retest reliability, the SwayDA-AP and SwayDA-ML tests were repeated after 5–7 days, at the same location. First, the Waterloo Footedness Questionnaire (Yang et al., 2018) was used to ascertain the dominant leg. For patients with LBP, the severity of pain was quantified by the numerical rating scale (NRS) (Karcioglu et al., 2018), and the impact of LBP on daily life was measured by the Oswestry disability index (ODI) (Chiarotto et al., 2016).

#### 2.3.3 Validity

Demographic information was collected first when the participants enrolled in the validity tests, and NRS, duration of pain, and ODI were collected in the LBP group. To avoid fatigue, The SwayDA tests were employed in the LBP group and asymptomatic control group before the mobility tests. Mobility was assessed in both groups using the 10 times sit-to-stand test, the 1-min sit-to-stand test (10-STS, 1 m-STS), and the lower extremity functional test (LEFT) (Shi et al., 2021). In the 10-STS, participants were asked to cross their hands on their chest, stand up straight, and then sit down, for 10 times. The maximum number of times that they could stand up from the seat in 1 min was recorded in the 1 m-STS. LEFT is a set of five functional tests, consisting of standing, turning, and hopping. The quality scores of both lower limbs in the LEFT were recorded separately. Specifically, the absolute value of the difference in the quality scores of both legs was then used in the validity test.

### 2.4 Data analysis

In order to obtain a bias-free measure of somatosensory acuity in voluntary postural sway, nonparametric signal detection theory was employed in the data processing of the SwayDA tests (Hautus et al., 2022). The probability of a correct response to the sway extent (true-positive judgment) and the probability of an incorrect response to the sway extent (false-positive judgment) were plotted as the receiver operating characteristic curve. Accordingly, the area under the receiver operating characteristic curve (AUC) was used to quantify the somatosensory acuity in the voluntary postural sway. AUC values range from 0 to 1, where 1 represents perfect somatosensory acuity and 0 represents an inability to discriminate between the different sway extents. Supplementary material 1 contains an illustrative example of data analysis for the SwayDA tests.

### 2.5 Statistical analysis

Continuous data were shown as mean ± standard deviation, including the results of NRS and ODI. Cronbach’s Alpha and the intraclass correlation coefficient (ICC) with 95% CI were utilized to reflect test–retest reliability. An ICC of 0.7 is defined as acceptable reliability (Post et al., 2016). A 95% confidence interval minimal detectable change was then calculated based on the standard deviation from the first test and ICC values (Steffen and Seney, 2008). Paired t-tests were conducted to find differences and determine consistency between the results of the first and the second visit for the SwayDA tests. In the known-groups validity and convergent validity tests, independent groups t-tests between the LBP group and the control group were used for the comparison of the AUC scores from the SwayDA tests and performance in mobility tests. To explore the relationship between performance in mobility tests and voluntary sway control ability, Pearson’s correlation test was used in the LBP group and the control group, respectively. A correlation coefficient from 0.3 to 0.5 suggests a moderate correlation, exceeding 0.5 indicates a strong correlation, while 0.1 to 0.3 implies a weak correlation (Cohen, 1988). Multivariate linear regression was then used based on the results of the correlation testing, where the dependent variables were the AUC values of the SwayDA tests, and the independent variables were the mobility tests. Covariates included the severity of pain and duration of pain. Statistical analysis was performed using SPSS version 26 for Windows (IBM, Seattle, United States), with the statistical significance set at *p* < 0.05.

## 3 Results

### 3.1 Test–retest reliability

Ten patients with LBP (age 21.6 ± 3.3 years, height 169.1 ± 6.2 cm and weight 61.1 ± 9.5 kg) and 10 age and sex-matched asymptomatic people (aged 24.8 ± 3.3 years, height 171.2 ± 8.6 cm, and weight 66.8 ± 7.8 kg) participated in this reliability test. The Cronbach’s Alpha of the SwayDA tests ranged from 0.743 to 0.798. The ICC (2.1) values of the SwayDA tests were over 0.7 which represented acceptable test–retest reliability (Table 1). There were no significant differences between the AUC values of the SwayDA tests at the first visit and the second visit (t_SwayDA-AP_ = 1.407, t_SwayAD-ML-D_ = -1.939, t_SwayDA-ML-ND_ = -0.284, all *p* > 0.05). In the LBP group, MDC_95_ of the SwayDA-AP, SwayDA-ML-D, and SwayDA-ML-ND tests were 0.071, 0.043, and 0.050, respectively.

**Table 1:** Cronbach’s Alpha and intraclass correlation coefficients (ICCs) for the AUC values of the SwayDA tests (mean ± SD).

		Low back pain group	Control group	Cronbach’s alpha	ICC (2.1)	95%CI
SwayDA-AP	First visit	0.736 ± 0.050	0.712 ± 0.07	0.750	0.741	(0.367, 0.896)
	Second visit	0.731 ± 0.024	0.688 ± 0.054			
SwayDA-ML-D	First visit	0.703 ± 0.029	0.69 ± 0.092	0.743	0.717	(0.309, 0.887)
	Second visit	0.718 ± 0.034	0.723 ± 0.071			
SwayDA-ML-ND	First visit	0.703 ± 0.040	0.721 ± 0.075	0.798	0.805	(0.502, 0.923)
	Second visit	0.714 ± 0.047	0.715 ± 0.06			

AUC, Area under the receiver operating characteristic curve (AUC). Higher AUC values represent better somatosensory acuity. SwayDA-AP, Sway discrimination apparatus test - anterior–posterior sway. SwayDA-ML-D, Sway discrimination apparatus test – medial-lateral sway to the dominant side. SwayDA-ML-ND, Sway discrimination apparatus test – medial-lateral sway to the non-dominant side. ICC, intraclass correlation coefficient.

### 3.2 Known-groups validity and convergent validity

Another 56 participants (28 patients with LBP and 28 control) were included in the validity test. The basic information of the two groups is shown in Table 2. There were no significant differences in age, sex, height weight (all *p* > 0.05).

**Table 2:** The basic information of the 56 participants for the validity tests (mean ± SD).

	LBP group	Control group
Number	28	28
Age (years)	19.5 ± 1.5	19.0 ± 1.1
Height (cm)	167.1 ± 7.7	166.9 ± 10.0
Weight (kg)	63.3 ± 12.6	62.5 ± 14
NRS	3.2 ± 0.7	/
Pain Duration	29.7 ± 15.4	/
ODI	9.3 ± 3.5	/

NRS, Numerical rating scale; ODI, Oswestry disability index.

#### 3.2.1 Voluntary postural control in patients with and without LBP

As shown in Table 3, significantly lower scores were found in the SwayDA-AP and SwayDA-ML-D tests (t_SwayDA-AP_ = –2.142, *p* = 0.037, Cohen’s *d* = −0.572; t_SwayDA-ML-D_ = –2.266, *p* = 0.027, Cohen’s *d* = −0.606, respectively) in the LBP group compared to the asymptomatic control group. Performance on the 1 m-STS was significantly worse in the LBP group compared to the control group (*t* = −2.313, *p* = 0.025, Cohen’s *d* = −0.618). However, there was no significant between-group difference in scores from the 10 times sit to stand test (*t* = 1.397, *p* = 0.168). The absolute error of the quality scores on the dominant side and the non-dominant side of LEFT in the LBP group was significantly higher than in the control group (*t* = 4.242, *p* < 0.001, Cohen’s *d* = 1.134), which indicated that the LBP group had significant bilateral differences in lower limb mobility compared to the control group.

**Table 3:** Comparison of the mean AUC values from the SwayDA tests and mean scores from the mobility tests, between the LBP and control groups (mean ± SD).

	LBP group	Control group	*t*	*p*	Cohen’s *d*
SwayDA-AP*	0.686 ± 0.088	0.726 ± 0.048	−2.142	0.037	−0.572
SwayDA-ML-D*	0.632 ± 0.053	0.661 ± 0.041	−2.266	0.027	−0.606
SwayDA-ML-ND	0.649 ± 0.066	0.666 ± 0.042	−1.111	0.271	−0.297
STS-1 min (number)*	52.6 ± 8.5	57.5 ± 7.2	−2.313	0.025	−0.618
STS-10 (seconds)	11.6 ± 1.6	11.0 ± 1.6	1.397	0.168	0.373
LEFT-AE**	6.4 ± 5.9	1.5 ± 1.6	4.242	<0.001	1.134

SwayDA-AP: Sway discrimination apparatus test - anterior–posterior sway. SwayDA-ML-D: Sway discrimination apparatus test – medial-lateral sway to the dominant side. SwayDA-ML-ND: Sway discrimination apparatus test – medial-lateral sway to the non-dominant side. 1mSTS: 1 min sit-to-stand test. STS-10: 10 times sit-to-stand test. LEFT-AE: The absolute error between the quality scores of the dominant side and non-dominant side of the lower extremities functional test.

*Significant difference at 0.05 level.

**Significant difference at 0.01 level.

#### 3.2.2 Relationship between the SwayDA tests, severity of LBP, and mobility tests

In Table 4A, it can be seen that a significant correlation was found between the SwayDA-AP, SwayDA-ML-D, and SwayDA-ML-ND in the LBP group (r_SwayDA-AP/ML-D_ = 0.553, r_SwayDAAP/ML-ND_ = 0.687, and r_SwayDAML-D/ML-ND_ = 0.681, all *p* < 0.01). Although no significant correlation was found between the SwayDA tests, NRS, and pain duration, the AUC values of the SwayDA-AP test were significantly correlated with ODI (r_AP-ODI_ = −0.391, *p* = 0.039) in the LBP group. Performances on the 1 m-STS and the 10-STS were significantly positively correlated with the AUC scores from the SwayDA tests (Table 4). Additionally, there was a significant negative correlation between the SwayDA tests and the absolute error between the quality scores of the dominant side and the non-dominant side in the LEFT test (−0.610 ≤ r ≤ −0.408, all *p* < 0.05). As shown in Table 4B, however, there was no significant correlation between the SwayDA tests and mobility tests in the control group.

**Table 4A:** Pearson’s Correlation between the SwayDA tests and mobility tests in 28 patients with LBP.

	SwayDA-AP	SwayDA-ML-D	SwayDA-ML-ND	NRS	Pain Duration	ODI	1 m-STS	10-STS	LEFT-AE
SwayDA-AP	1	0.553**	0.687**	−0.227	0.093	−0.391*	0.384*	−0.201	−0.449*
SwayDA-ML-D		1	0.681**	−0.333	−0.004	−0.211	0.441*	−0.513**	−0.408*
SwayDA-ML-ND			1	−0.214	0.085	−0.318	0.418*	−0.420*	−0.610**

SwayDA-AP: Sway discrimination apparatus test - anterior–posterior sway. SwayDA-ML-D: Sway discrimination apparatus test – medial-lateral sway to the dominant side. SwayDA-ML-ND: Sway discrimination apparatus test – medial-lateral sway to the non-dominant side. NRS: Numerical rating scale. ODI: Oswestry disability index. 1 m-STS: 1-min sit-to-stand test. 10-STS: 10 times sit-to-stand test. LEFT-AE: The absolute error between the quality scores of the dominant side and non-dominant side of the lower extremities functional test.

* Correlation is significant at the 0.05 level (2-tailed).

** Correlation is significant at the 0.01 level (2-tailed).

**Table 4B:** Pearson’s Correlation between the SwayDA tests and mobility tests in asymptomatic controls.

	SwayDA-AP	SwayDA-ML-ND	SwayDA-ML-D	1 m-STS	10-STS	LEFT_AE
SwayDA-AP	1	−0.205	0.248	0.222	−0.304	0.202
SwayDA-ML-ND		1	−0.106	0.194	−0.151	−0.113
SwayDA-ML-D			1	−0.263	0.125	0.004

Based on the correlation results, further analysis via multivariate linear regression was carried out. The results show that AUC values of the SwayDA-AP test and SwayDA-ML-ND test could be significantly predicted by ODI, the absolute error in LEFT, and score on the 1 m-STS test (F_AP_ = 7.714, *p* = 0.001, Adjusted *R*^2^ = 0.427; F_MLND_ = 13.583, *p* < 0.001, Adjusted *R*^2^ = 0.583). A significant association was also found between the absolute error in LEFT, 1 m-STS, and SwayDA-ML-D test scores (F_MLD=_6.534, *p* = 0.005, Adjusted *R*^2^ = 0.291). When covariates, the severity of pain and duration of pain, were included in the multivariate linear regression model, there were no alterations observed in the previously obtained results. The process and formula of the multivariate regression were shown in Supplementary material 2.

## 4 Discussion

In this study, we aimed to investigate the reliability and validity of an evidence-informed and modified apparatus (Chen et al., 2019) for the quantitative evaluation of somatosensory acuity in voluntary postural sway in a population of asymptomatic and LBP individuals. The Cronbach alpha coefficient and ICC results both showed that the SwayDA tests had acceptable test–retest reliability. In the difference analyses, the LBP group had significantly lower somatosensory acuity scores during voluntary anteroposterior sway and mediolateral sway to the dominant side, compared to the asymptomatic controls. There was a significantly poorer performance on the 1 m-STS test in the LBP group, and the bilateral differences observed in lower limb mobility in the LBP group were significantly larger than those in the asymptomatic control group. With regard to the convergent validity test, the results demonstrated strong pairwise correlations between the SwayDA-AP, SwayDA-ML-D, and SwayDA-ML-ND tests in the LBP group, a result which was consistent with the side-general effect previously noted in the quality of proprioceptive information (Han et al., 2013).

Some studies have shown that patients with LBP have altered postural adjustment strategies and performance in voluntary postural tasks (Azadinia et al., 2023; Rum et al., 2023). In this study, the LBP group showed lower AUC values on the SwayDA tests and performance in the mobility tests, and there was a positive linear association between the SwayDA tests and mobility tests. Interestingly, no significant differences were found between the two groups in the AUC values from the SwayDA-ML-ND test, suggesting that somatosensory acuity during mediolateral postural sway to the non-dominant side was likely less affected by LBP. A possible explanation may be made by referring to the side general effect in lower limb proprioception, where Han et al. found that proprioception of the non-dominant side was significantly better than that of the dominant side (Han et al., 2013). Due to the laterality of lower limb proprioception, the pattern in the SwayDA tests here also showed a similar side effect, in that the somatosensory acuity of the dominant side was more affected by LBP than that of the non-dominant side.

To prevent a ceiling effect in the data, challenging mobility tests were selected for use in this study, such as 1 m-STS and LEFT tests, based on the demographic characteristics of the included participants (Zemková and Hamar, 2018). In the LEFT test, we found that differences in mobility between the lower limbs in patients with LBP were significantly higher than in asymptomatic controls. The differences in mobility between bilateral lower limbs may cause an imbalance in the spinal stability structure, which may be one of the causes of LBP (de Sousa et al., 2019). Furthermore, the present study showed a significant moderate to strong level of negative correlation between the AUC values in the SwayDA tests and bilateral differences of LEFT in the LBP group. Combining the side effects of somatosensory decline, patients with LBP may rely more on the somatosensory perception arising from one side of the body, and consequently, the imbalance in mobility between the lower limbs may then worsen.

A significant moderate negative correlation was found between ODI and the SwayDA-AP test, though there was no significant association between the severity of pain, duration of pain, and the SwayDA tests. Through further linear regression, we found that 29.1–58.3% of the variation of the AUC scores for the SwayDA in the LBP group could be explained by 1 m-STS, LEFT, and ODI scores. Compared to young adults with LBP, the somatosensation and motor control in elderly patients with LBP may have been remodeled by long-term pain and aging (Sakai et al., 2022). Some studies have shown a synchronous decline in somatosensory acuity, mobility, and activities of daily living in the elderly with LBP (Rudy et al., 2007; Rundell et al., 2015; Xiao et al., 2022). In young adults with LBP, however, the decline in somatosensory acuity and mobility associated with pain may still be compensated, because young patients have a shorter duration of pain and a higher baseline of physical function compared to the elderly with LBP (GBD, 2021). The participants included in this study were young adults. Therefore, although there was significantly lower somatosensory acuity and mobility in the LBP group, these changes may not be linearly related to the severity and duration of pain.

### 4.1 Strength and limitations

To the best of the authors’ knowledge, the SwayDA is the first device designed for the evaluation of somatosensory acuity during voluntary postural sway. Previous studies have concentrated on assessing regional proprioception via customized devices or isokinetic systems, such as the lumbar spine proprioception (Tong et al., 2017; Lin et al., 2019). The proprioception assessments may not be readily available in clinical settings. In contrast, the SwayDA was designed with a focus on high ecological validity, because the SwayDA mimicked voluntary weight shifting, which is a common aspect in activities of daily living, such as sit-to-stand or walking. The results of this study suggest that somatosensory acuity in voluntary postural sway can be inferred from commonly used functional tests and LBP-related questionnaires, applied in a clinical setting.

While the validity of the present novel technology needs to be explored further before wider clinical application, the limitations of its clinical applicability can be compensated for through the use of simple and acceptable clinical tests and scales. Considering the significant correlation between somatosensory sensitivity and lower limb mobility, we plan to investigate the influence of lower limb proprioception and muscle strength on somatosensory perception in future research. The participants included in this study were young (mean age = 19.5 ± 1.5 years) with minimal pain (mean NRS = 3.2 ± 0.7). Therefore, this limits the applicability of our study to the general LBP population (older and higher pain severity). Finally, this pilot study used a pragmatic sample size to demonstrate the novel and reliable methodology for assessing somatosensory acuity during voluntary postural sway. In the future, a larger-scale study involving a wider array of LBP severity and age groups is needed to improve the generalizability of the findings.

## 5 Conclusion

The modified SwayDA tests conducted in the coronal and sagittal planes demonstrated acceptable reliability in quantitatively evaluating somatosensory acuity during voluntary postural sway for adults with LBP. In the known-groups validity test, patients with LBP exhibited lower somatosensory acuity and mobility, and an imbalance of mobility between their lower limbs. Poorer somatosensory acuity was also significantly correlated with higher LBP-related disability. Impaired somatosensory acuity was also significantly linked to diminished mobility and bilateral mobility imbalance. Future studies could include patients with LBP with ages across the full lifespan to enhance the generalizability of the findings and to explore the relationship between lower limb functions, including proprioception and muscle strength, and somatosensory acuity, during voluntary postural control. This will inform personalized rehabilitation intervention to optimize recovery and injury prevention.

## Data availability statement

The raw data supporting the conclusions of this article will be made available by the authors, without undue reservation.

## Ethics statement

The studies involving humans were approved by Swinburne University of Technology Human Research Ethics Committee (Ethics ID: 20225788-11032). The studies were conducted in accordance with the local legislation and institutional requirements. The participants provided their written informed consent to participate in this study.

## Author contributions

ZC: Conceptualization, Data curation, Formal analysis, Investigation, Writing – original draft. OT: Data curation, Software, Supervision, Writing – review & editing. JH: Conceptualization, Funding acquisition, Methodology, Resources, Supervision, Writing – review & editing. RA: Methodology, Writing – review & editing. DE-A: Conceptualization, Project administration, Supervision, Writing – review & editing. AP: Conceptualization, Data curation, Formal analysis, Supervision, Writing – review & editing.
