# Supplementary material for: Voluntary postural sway control and mobility in adults with low back pain
Source: Front Neurosci. 2024 Jan 3;17:1285747. doi: 10.3389/fnins.2023.1285747 (PMC10793656; doi:10.3389/fnins.2023.1285747)
Supplement: Supplementary file 1 [file Presentation_1.pdf]

Supplement 1: Procedure and data analysis of the SwayDA tests

<u>The testing procedure of the SwayDA</u>

1. Preparation

The Sway Discrimination Apparatus (SwayDA) tests comprised three types: the SwayDA test for anteroposterior postural sway (SwayDA-AP test), mediolateral sway to the dominant side (SwayDA-ML-D test), and mediolateral sway to the non-dominant side (SwayDA-ML-ND test). The order of these three types of the SwayDA tests was random. The estimated duration for each of the SwayDA tests was approximately 5 minutes. Participants were given a one-minute break to avoid fatigue before the commencement of each test. Before the SwayDA tests, participants were required to wear sports clothes. The SwayDA tests did not employ headphones or blindfolds to enhance ecological validity.

2. The SwayDA-AP test

In the SwayDA test for anteroposterior postural sway (SwayDA-AP), there is a scale on one of the wooden stops, which provides a set of 4 predetermined anterior-posterior sway extents (Figure 1a). Four sway amplitudes were pre-set on the movable stop. Position 1 was set 2 cm in front of the anterior superior iliac spine (ASIS), and Positions 2, 3, and 4 represented 2.5 cm, 3 cm, and 3.5 cm anteroposterior sway distance, respectively. The wooden stop on the opposite side served as a reference point for the neutral standing position for participants in the SwayDA-AP test.

- **Familiarization Session**

  Before data collection, a familiarization session was conducted, comprising 3 rounds of practice from Position 1 to Position 4 sequentially (a total of 12 practice trials). Participants were instructed on the pre-determined 4 positions during the familiarization session. Facing the movable stop on one side, the participants were instructed to look straight ahead without a fixed target as participants may obtain additional visual cues from a fixed target[1]. The two wooden stops were adjusted to the level of participants' ASIS. Then participants were required to sway forward using their ankles whilst keeping their hips and knees fixed, like an inverted pendulum model. After their ASIS contacts the wooden stop, participants should sway back to the neutral starting position.

- **Data Collection Session**

  During the data collection session, the 4 sway amplitudes were presented 10 times in a random order, with 40 trials in total in the SwayDA-AP test. During each trial, participants were required to make an appropriate numeric judgment (Position 1, 2, 3, or 4) to the extent that they just swayed, as soon as they returned to the starting position. The numeric judgments were recorded for further analysis.

3. The SwayDA-ML tests

For the SwayDA-ML tests, a different wooden stop (as shown in Figure 1b) was designed for mediolateral sway with Position 1 representing 4 cm from the greater trochanter of the femur on the test side, and Positions 2, 3, and 4 were set as 4.5 cm, 5 cm, and 5.5 cm, respectively. The wooden stop on the opposite side served as a reference point of neutral standing position for participants in the SwayDA-ML tests.

- **Familiarization Session**

  The familiarization session in the SwayDA-ML tests followed a consistent process with the SwayDA-AP test. This session assisted participants in becoming familiar with the four pre-set voluntary mediolateral sway extents, designated as Positions 1 to 4. Participants were instructed to stand on the testing platform with their heels aligned with the ischial tuberosities, and they were directed to clasp their hands in front of their bodies. When standing, participants were instructed to gaze straight ahead without any reference markers in their field of vision. Subsequently, the wooden stops on both sides were adjusted to the level of the greater trochanter. During the familiarization session, participants were instructed to voluntarily sway until the greater trochanter lightly touched one wooden stop, and then immediately return to the neutral standing position by touching the other wooden stop. Voluntary mediolateral sway was like an inverted pendulum model, initiating from the ankle joint and maintaining alignment between the body and lower limbs (not hip tilting). In the SwayDA-ML-D test, the sway direction is the dominant side, while in the SwayDA-ML-ND test, the sway direction is the non-dominant side.

- **Data Collection Session**

  This session maintained consistency with the SwayDA-AP test process. The participants were required to report the position number, as soon as they swayed

back to the neutral standing position. Each of the 4 mediolateral sway extents was randomly presented 10 times, with 40 trials in total in the SwayDA-ML-D test and the SwayDA-ML-ND test. The 40 responses from the participants were recorded in a table for data processing.

Data analysis

To quantify a bias-free measure of somatosensory acuity, the signal detection theory was used to process the data [2]. During data processing, the probability of correct answers to the stimuli (Position 1 to 4) was treated as "hit" or true-positive judgment, while the probability of incorrect answers to the sway amplitudes was regarded as "false alarm" or false-positive judgment. The true-positive in the y-axis and false-positive judgment in the x-axis were then plotted as the receiver operating characteristic (ROC) curve. The area under the ROC curve (AUC) scores were considered as a measure to reflect how accurately a participant can discriminate between Position 1, 2, 3, and 4. The AUC has both upper (1.0) and lower (0.0) boundaries where 1.0 represents perfect somatosensory acuity during voluntary postural sway, 0.5 is chance responding, and 0.0 means inability to correctly judge the differences between stimuli. The statistical procedure is as follows:

(1) The raw data of the 40 trials from the participants were first recorded. As shown in Table S2.1, the second column represents stimuli (Positions 1 to 4), and the third column represents responses from the participants (1 to 4). The order of stimuli was random.

Table S2.1 The raw data sheet. The second column represents the randomized order of the 4 pre-set stimuli. The responses from a participant were recorded in the third column.

Trial	Position	Response
1	4	2
2	3	1
3	2	1
4	2	2
…	…	…
…	…	…
37	1	1
38	2	2
39	4	4

40	1	1

(2) The frequency of the responses (Table S22) to the four different stimuli was converted from the raw data table.

Table S2.2 Converted frequency table for different stimuli (Position 1 to 4). The sum of frequencies per row is always 10, as each position randomly appeared 10 times in the SwayDA tests.

	Response			
Position	1	2	3	4
1	8	2	0	0
2	5	4	1	0
3	1	3	4	2
4	0	2	4	4

(3) In the SwayDA tests, there were three pairwise comparisons for the presence of four different stimuli, namely Position 1 vs. Position 2, Position 2 vs. Position 3, and Position 3 vs. Position 4. For example, when comparing responses to Position 1 and Position 2, the cumulative frequencies of the participants' responses to Position 1 and Position 2 were generated (Table S2.3).

Table S2.3 The cumulative frequency for Position 1 and Position 2.

	Response			
Position	1	1 and 2	1, 2, and 3	1, 2, 3, and 4
1	8	10	10	10
2	5	9	10	10

(4) To draw the ROC curve, cumulative probabilities were then calculated based on the cumulative frequencies. In the example of Position 1 vs. Position 2, the likelihood of a participant responding with a "1" to Position 1 was 80%, and the probability increased to 100% when the participant responded to Position 1 with either "1" or "2" (Table S2.4).

Table S2.4 The cumulative probabilities for Position 1 and Position 2.

	Response			
Position	1	1 and 2	1, 2 and 3	1, 2, 3 and 4
1	0.8	1	1	1
2	0.5	0.9	1	1

(5) The ROC curve was then determined based on the cumulative probability table (See Figure 3.3). In this case of Position 1 vs. Position 2, the probability of true-positive judgment (responding with a "1" to Position 1) was 0.8 based on the signal detection theory, and false-positive judgment (responding with a "1" to Position 2) was 0.5. When 'hit' was regarded as a response with “1” and “2” to Position 1, the true positive rate was 1.0, and accordingly, the false positive rate was 0.9. The AUC was then computed by summing the component areas as 0.660 (the shed part of Figure S2.1).

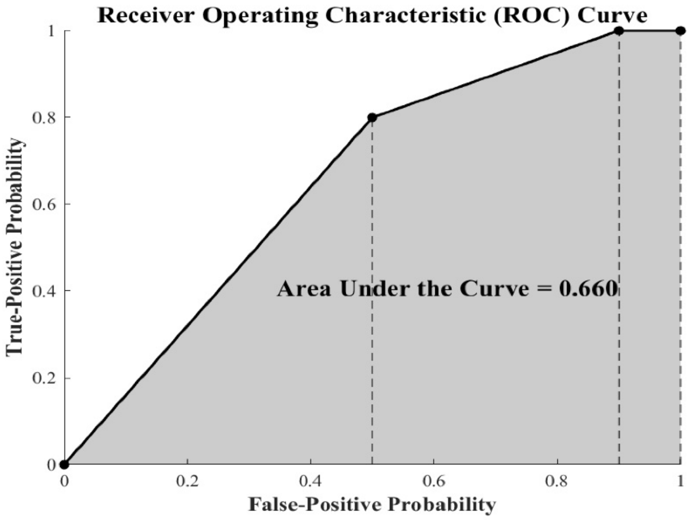

Figure S2.1 The Receiver Operating Characteristic (ROC) Curve for discrimination ability between Position 1 and 2 in a participant. The shaded area represents the area under the ROC curve (AUC).

(6) Then the AUC values of Position 2 vs. Position 3 and Position 3 vs. Position 4 were calculated as 0.815 and 0.650, respectively. Finally, the average AUC value for this participant was (0.660+0.815+0.650)/3=0.708, indicating the participant’s

ability to discriminate between the 4 preset sway amplitudes was better than random guessing, but not yet perfect.

References

[1] R. Moraes, P.B. de Freitas, M. Razuk, and J.A. Barela, Quality of Visual Cue Affects Visual Reweighting in Quiet Standing. PLoS One 11 (2016) e0150158.

[2] M. Hautus, N. Macmillan, and C. Creelman, Detection Theory: A User's Guide, 3rd ed., Routledge, New York, 2022.
