# Supplementary material for: Voluntary postural sway control and mobility in adults with low back pain
Source: Front Neurosci. 2024 Jan 3;17:1285747. doi: 10.3389/fnins.2023.1285747 (PMC10793656; doi:10.3389/fnins.2023.1285747)
Supplement: Supplementary file 2 [file Presentation_2.pdf]

Supplement 2. The process and results of linear regression between the SwayDA tests and mobility tests.

The purpose of this multivariate linear regression was to clarify whether the area under the curve (AUC) values of the sway discrimination apparatus (SwayDA) tests could be predicted by numeric rating scale (NRS), Oswestry disability index (ODI), and mobility tests. Mobility tests included 1-minute sit-to-stand test (1m-STS) and lower extremity functional test (LEFT). The absolute value of the differences in the quality scores of both sides in LEFT (LEFT-AE) was used in the regression, which represented the difference in mobility of both lower limbs. Ten times sit-to-stand test (10-STS) was excluded from the regression considering the problem of multicollinearity between 1m-STS and 10-STS. Those independent variables with a significance level less than 0.1 in Pearson's correlation would be included in multivariate linear regression analysis using the backward elimination technique. Covariates included the severity and duration of pain. The Durbin-Watson statistic was calculated to reflect the multicollinearity among the included independent variables. Since there was no significant association between the SwayDA tests and mobility tests in the control group, the following results only involve the Low back pain group (28 participants)

1. The SwayDA-AP test as the dependent variable

F test showed that there was a significant association between the SwayDA-AP test and 1m-STS, ODI, and LEFT-AE (F=7.714, p=0.001, Adjusted R^2 = 0.427) with Durbin-Watson = 2.656.

The t-test results of each independent variable were shown in Table S1.

Table S1. The t-test results of the independent variables in the SwayDA-AP test

	t	p	β
LEFT-AE	-2.997	0.006	-0.006

ODI	-2.733	0.012	-0.010
1m-STS	2.479	0.021	0.004

The formula was as followed:

SwayDA-AP = 0.623 -0.006 * LEFT-AE - 0.010 * ODI + 0.004 * 1m-STS

2. The SwayDA-ML-D test as the dependent variable

The SwayDA-ML-D test was significantly associated with 1m-STS and LEFT-AE (F=6.534, p=0.005, Adjusted R^2 = 0.291). There was an acceptable serial correlation between the independent variables as the Durbin-Watson statistic was equal to 2.103. The t-test for 1m-STS and LEFT-AE were shown in Table S2.

Table S2. The t-test results of coefficients in the regression model between the SwayDA-ML-D tests and mobility tests

	t	p	β
LEFT-AE	-2.381	0.025	-0.003
1m-STS	2.596	0.016	0.003

The formula was as followed:

SwayDA-ML-D = 0.517 - 0.003 * LEFT-AE + 0.003 * 1m-STS

3. The SwayDA-ML-ND test as the dependent variable

The results of the F test showed a significant relationship between the SwayDA-ML-ND test and 1m-STS, ODI, and LEFT-AE (F=13.583, p<0.001, Adjusted R^2 = 0.583). The Durbin-Watson test showed an acceptable serial correlation among the independent variables (Durbin-Watson = 2.200).

Table S3. The results of coefficients of the independent variables in the SwayDA-ML-ND test

	t	p	β
LEFT-AE	-4.788	<0.001	-0.007
ODI	-2.632	0.015	-0.006
1m-STS	3.112	0.005	0.003

The formula was as followed:

SwayDA-ML-ND = 0.590 - 0.007 * LEFT_AE + 0.003 * 1mSTS -0.006 * ODI
